# 

**Published:** 1888-03

**Authors:** 


SANDERS & SONS' EUCALYPTI EXTRACT (*Eucalyptol*).—Apply to Dr. Sanders, Dillon, Iowa, for gratis supplied reports on cures effected at the clincs of the Universities of Bonn and Griefswald.

